# Establishing and sustaining women-led vector control groups: community perspectives from Misungwi and Ilemela districts, Mwanza, Tanzania

**DOI:** 10.1186/s41936-025-00448-3

**Published:** 2025-05-05

**Authors:** Basiliana Emidi

**Affiliations:** National Institute for Medical Research Dodoma Centre, Dodoma, Tanzania

**Keywords:** Community perspectives, Women in vector control, Vector-borne diseases, Mosquito, Malaria, Dengue, Trainers of Trainees, Mwanza, Tanzania

## Abstract

**Background:**

Vector-borne diseases (VBDs), specifically mosquito-borne diseases, are widespread in most sub-Saharan Africa countries. These diseases place significant strain on public health systems across the region. Community engagement has long been recognized as a key component of public health interventions. Since the Alma-Ata Declaration in 1978, efforts have been made to involve communities in healthcare programmes. Strengthening the community engagement strategies is essential for the success of malaria control and other mosquito-borne diseases. Women are key change agents in the efforts to combat VBDs. Involving them in the VBDs control and management efforts increases the levels of community acceptance and compliance. This qualitative study employed in-depth interviews (IDIs) and focus group discussions (FGDs) to explore community perspectives on establishing and sustaining women-led vector control groups. Data were collected from community members. Understanding community support for women’s involvement in vector control is key to designing effective and sustainable programmes. This study aimed to assess the community perspectives and support for establishing women groups working in vector control at community level.

**Results:**

The study findings revealed that many of the respondents had a positive perception towards and accepted the idea of establishing and sustaining the ‘women in vector control’ groups at the community level. Most respondents agreed that these groups of women would help educate other community members on the importance of keeping their environment clean. They pointed out that women have a unique ability to pass such information passionately and persuasively to other people. A few respondents, however, were sceptical of this initiative. They believed that women occupy a subordinate social position in society—with little or no privilege and authority—thus making it hard for them to participate in the public activities. Most respondents also indicated that the community would support and sustain this group of women through provision of incentives.

**Conclusion:**

Communities accept and support the idea of establishing and sustaining ‘women champions in vector control’ groups to eliminate VBDs. The study highlights the potential of involving women in vector control efforts as a community-driven strategy to improve public health, with strong support for the initiative and recognition of women’s effectiveness in health education.

## Background

Vector-borne diseases (VBDs) account for over 17% of all infectious diseases, leading to more than 700,000 deaths annually (WHO, 2017). Mosquitoes are primary vectors, transmitting malaria, dengue, chikungunya and lymphatic filariasis (WHO, 2020). In sub-Saharan African countries (SSA), vector-borne diseases (VBDs) are endemic and exert enormous pressure on the public health systems (WHO, 2017). The SSA is the most affected region, whereby four countries accounted for over half of all malaria deaths globally: Nigeria (31.9%), the Democratic Republic of the Congo (13.2%), the United Republic of Tanzania (4.1%) and Mozambique (3.8%) (WHO, 2021). Dengue fever affects approximately 390 million people each year, with over 5 million cases and 5000 deaths reported in 2023 (WHO, 2023). To tackle the burden of vector-borne disease, various vector control interventions have been implemented, including the use of insecticide-treated nets, indoor residual spraying and environmental management (Bhatt et al., 2015). However, for long-term sustainability, both national vector control programmes and the global vector control response have emphasized the crucial role of community engagement and mobilization (WHO, 2017; MoHCDGEC, 2019). Community engagement has been recognized as an important element of public health since the Alma-Ata declaration in (1978), whereby efforts have been made to involve communities in health care programmes (Robertson, 1978).

However, practicing community engagement has played a marginal role within malaria control programmes (Geissbühler, 2008; Whittaker & Smith, 2015). Empowering communities to participate in vector control efforts enhances interventions effectiveness, promotes behavioural change and ensures the long-term success of disease prevention strategies (Carboni et al., 2023).

Re-conceptualize and better strategize community engagement, has become increasingly important for malaria control and elimination programme success (Akogun et al., 2001). Previous studies on women involvement in vector control conducted in Rwanda, Madagascar, Zambia and Mali have documented that women are key change agents in the effort to combat VBDs (Donner et al., 2017; Shiras et al., 2023). Therefore, their involvement in the design, leadership and execution of health interventions increases the levels of community acceptance and compliance and help shape best practices in VBD control and elimination (Mungall-Baldwin, 2022). The active participation of women in vector control activities has multiple benefits, including equal access to employment opportunities, improved health outcomes and sustainability of vector control programmes (Donner et al., 2017; Gunn et al., 2018; Shiras et al., 2023). Women have demonstrated specific qualities aiding successful implementation of various programmes, therefore empowering them and assessing the community acceptability, and perception is paramount (Mungall-Baldwin, 2022). Evidence has shown that engaging women in vector control can increase acceptability and sustain activities in their communities (Gunn et al., 2018). Despite these benefits, gender equality in vector control programmes has not been fully realized. Many initiatives lack deliberate strategies to integrate women into leadership and decision-making roles (Donner et al., 2017). Few programmes have designed and assessed policies aimed at promoting gender equity in vector control. Addressing these gaps is essential for enhancing programme effectiveness and sustainability. Training and awareness creation on women’s role in vector control are crucial. Empowering women with information on environmental management, mosquito net distribution and other vector control measures helps them to take action. It also builds confidence and fosters a sense of control over health-related issues that affect the lives of community members (Tapia-Conyer et al., 2012; Ng’ang’a et al., 2021). One strategy to support women in vector control activities is the establishment of women-led groups who will be the trainers of trainees and can provide supportive supervision regarding vector control in their communities. Therefore, the present study aimed to assess the community perspectives on the establishment and sustainability of women-led vector control groups at community level.

## Methods

### Study design and setting

**Fig. 1:**
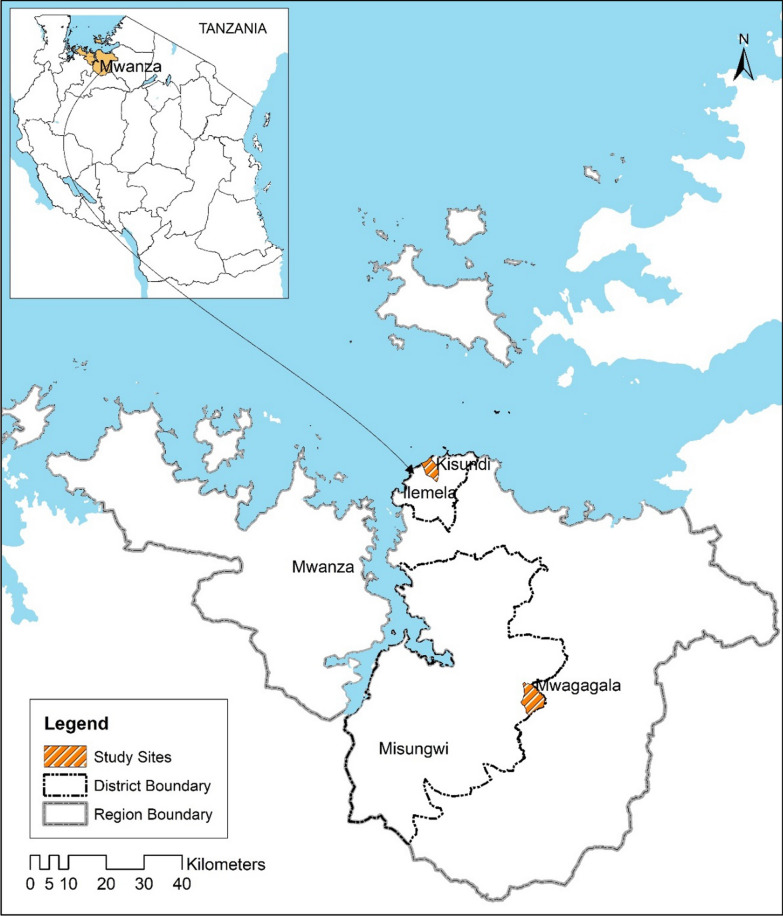
Map of Mwanza region showing the study sites

The present study was implemented at Misungwi and Ilemela districts in Mwanza region, Tanzania. Misungwi district is located at 2°34.673′S and 33°07.170′ E and covers an area of 2122 km^2^. The annual rainfall ranges from 0.5 mm to 58.8 mm, split in two rainy seasons (October to December and March to May). Ilemela district is one of the seven districts of the Mwanza Region of Tanzania. Ilemela district is located at 02°35′S and 32°55′E. It is bordered to the north and west by Lake Victoria, to the east by Magu District, and to the south by Nyamagana District (URT, 2022). The economic activities of the residences of Misungwi are subsistence farming and livestock keeping. The main malaria vectors mosquitoes in Misungwi district are *Anopheles funestus* and *Anopheles gambiae complex* and are predominant during the rainy season. Malaria vectors are resistant to pyrethroids insecticides in the area. In each district, two sites, Kisundi and Mwagagala, were selected from Ilemela and Misungwi, respectively (Fig. 1).

### Study design

This was a cross-sectional qualitative study that used focus group discussions (FGDs) and in-depth interviews (IDIs). Participants selected included the adult and youth men and women, village and cultural leaders. The cultural leaders, including traditional chiefs, elders, religious leaders and community influencers, play a crucial role in shaping public perceptions, mobilizing community support and facilitating behaviour change (Vilas-Boas et al., 2018). The study participants were interviewed to obtain information on their perceptions, views, acceptability and support towards establishing and sustaining a group of women who will serve as trainers of trainees (TOTs) in terms of vector control activities in their community.

### Sampling and recruitment

The selection of the study respondents was based on inclusion criteria such as being residence in the selected village and street, physically and mentally fit, with 18 and above years old. The study participants’ identification and recruitment were done with the help of the village leaders. Village leaders, being well-acquainted with community members, provided insights into the demographic and social characteristics of potential participants, ensuring that the selected individuals were representative of the community. Prior to the interviews, study participants consented voluntarily to participate in the study.

### Data collection and sample size

Evidence has indicated that the point of saturation can be reached after conducting 10–17 IDIs, and four–eight for focused group discussions (Hennink & Kaiser, 2022). Therefore, for this study a total of 37 IDIs and nine FGDs were conducted. Each FGD comprised of six to 12 respondents. Guides for the interview and FGDs aimed to assess knowledge on mosquito vector control, acceptability, perception and support for establishing and sustaining ‘women in vector control’ groups.

### Data management and analysis

Data collected through in-depth interviews (IDIs) and focus group discussions (FGDs) were transcribed verbatim and stored securely, with personal identifiers removed. The transcripts were analysed using thematic analysis, starting with familiarization, followed by coding, and grouping of codes into broader themes related to the study objectives. The research team interpreted the data by identifying patterns and relationships to address the research questions, supported by participant’s quotes. Triangulation of IDI and FGD data was used to validate findings. Data analysis was conducted iteratively, ensuring transparency, consistency and alignment with ethical guidelines to protect participant confidentiality.

### Ethical considerations

The present study has been approved by the Lake Zone Institutional Review Zonal Board (LZIRB) of the National Institute for Medical Research (NIMR), Tanzania, with reference number MR/53/100/761. The permission to publish has been granted by NIMR with reference number BD.242/437/01C/83.

## Results

### Socio-demographic characteristics

A total of 125 respondents were interviewed. Among them, 77 were from Kisundi street and 48 from Mwagagala village. Majority of respondents aged between 18 and 30 years. Among the respondents interviewed, female accounted to large proportion in both sites. Majority of respondents were married and had primary education. Large proportion of respondents were farmers (Table 1).

**Table 1:** Socio-demographic characteristics of the respondents

Characteristics	Total, *n* (%) Mwagagala	Total, *n* (%) Kisundi
Age		
18–30	19 (39.6)	27 (35.1)
31–40	7 (14.6)	21 (27.3)
41–50	18 (37.5)	21 (27.3)
51–60	3 (6.3)	5 (6.5)
60 +	1 (2.1)	3 (3.9)
Sex		
Male	22 (45.8)	34 (44.2)
Female	26 (54.2)	43 (55.8)
Education level		
Never attended	4 (8.3)	5 (6.5)
Primary	41 (85.4)	67 (87.0)
Secondary	3 (6.3)	4 (5.2)
University	0 (0.0)	1 (1.3)
Marital status		
Single	8 (16.7)	17 (22.1)
Married	38 (79.2)	56 (72.7)
Divorced	2 (4.2)	3 (3.9)
Widow/widower	0 (0.0)	1 (1.3)
Occupation		
Farmer	42 (87.5)	73 (94.8)
Health officer	1 (2.1)	1 (1.3)
Agriculture officer	1 (2.1)	0 (0.0)
Veterinary officer	0 (0.0)	0 (0.0)
Village/sub-village/cultural leaders	4 (8.3)	3 (3.9)

### Community understanding on mosquitoes and their breeding sites

During FGD and IDI conversations, valuable insights about mosquitoes and their breeding sites were pointed out. The findings have revealed that participants were knowledgeable about the common habitats where mosquitoes breed, such as stagnant water, ponds, and containers. They further pointed out various environments such as water, puddles, swamps, ponds, and dark places where mosquitoes can breed. They associate mosquitoes with unclean environments, particularly dirty places, and grassy areas (areas with abundant grasses) puddles, tree holes, trash, riverbanks, dark places, and bathrooms. Both male and female participants, demonstrated an adequate understanding of mosquitoes and their breeding sites. Many participants were able to correctly mention specific areas in their surroundings where mosquito larvae could be found. For instance, one participant mentioned that,

“*Although mosquitoes can be found anywhere, they tend to be located near stagnant water sources especially during the rainy season. When people are living near such water sources like ponds, they are at a higher risk of being bitten by infected mosquitoes and contacting the malaria.*” (FGD-R5-female adult).

### Health risks associated with mosquito bites

The findings of the present study have indicated that participants were generally aware of the health risks associated with mosquito bites, with a primary focus on malaria as the most significant health threat. The participants demonstrated a clear understanding that mosquito bites can lead to serious illnesses, particularly malaria, with many noting symptoms such as fever, chills and fatigue. For example, one participant stated,

“*For what I understand, mosquito is an insect which causes diseases.”* (FGD-R8-male.adult).

It was mentioned that getting infective mosquito bites can result in health risks to people especially pregnant women and children as it was pointed out by one woman that,

*“Malaria-carrying mosquitoes can also be a threat to expecting mothers. In case a pregnant woman is bitten by mosquito carrying malaria, it can result in a pregnancy loss.*” (FGD-R4-female adult).

Some participants also mentioned the risk of death associated with malaria and ill-conditions such as stomach pain, headache and fever. Other health risks mentioned included seizures in children, rashes, headaches, confusion, vomiting, weakness and hallucinations.

### Community views on the control of mosquitoes

The findings of this study have revealed that participants have a good understanding of various mosquito control methods aimed at eliminating mosquito vectors that carry malaria parasites. Among the control measures mentioned, included cleaning the environment, clearing bushes, filling water holes, cutting grass and avoiding staying near water bodies. Both male and female participants demonstrated similar knowledge on these methods. Several participants narrated,

*“We can minimize mosquito breeding ground*” (FGD-R2-male adult) and

“*Cleaning the environment”.* (FGD-R8-male adult),

*“As evening approaches, it is advisable for individuals to shut their door order to prevent mosquitoes from entering in the house”.* (IDI-R8-female adult)

“*Covering the holes near the house, to cut the trees near the house, mowing the glass, and spraying*”. (FGD-R4-female youth)

*“Closing the door when you’re about to sleep*”. (FGD-R2-female youth)

This suggests a shared understanding of the importance of eliminating breeding sites to control mosquito populations.

Regarding malaria prevention, participants showed a high level of awareness regarding behaviours to protect themselves from mosquito bites. Measures such as using mosquito nets, applying mosquito repellents and wearing long-sleeved clothing, while both men and women understood the importance of these actions. However, women were more likely to report consistent use of mosquito nets, due to their caregiving roles in their families. A female participant shared,

“*At night, we ensure our kids remain indoors as we are conscious that malaria-carrying mosquitoes can be present at any time. To safeguard against this, we use mosquito nets at night”* (FGD-R2-female adult)

Participants highlighted the evolving approach to malaria treatment and the role of women in influencing health-seeking behaviours within their communities. The participants reflected on the importance of consulting healthcare professionals in health facilities before administering medication. This highlights a growing awareness of the need for accurate diagnosis rather than relying on assumptions. This transformation in health practices within the community illustrates how women are integral in shaping and promoting better health outcomes, particularly in the context of malaria prevention and treatment. As narrated by a female participant that,

“*As a woman, if my child gets malaria, I will rush him to the hospital for treatment. After being given medication, he will recover. Then, if I hear that my neighbour’s child is sick, I will advise her to take the child to the hospital. In our community, we have been adapted to buying medicines without knowing if the child is sick of malaria or not, but nowadays we cooperate to give advice and take a child to the health facility for diagnosis before give the child medication.”* (FGD-R7-female adult)

### Community needs for mosquito control expertise

The lack of empowerment at community level to have expertise on vector control was seen as a gap which need to be addressed. Therefore, empowering women on vector control at community level is paramount. During discussion, it was evident that respondents need skills on how to identify the mosquito breeding ground and destroy them as pointed out that by one respondent that,

“*We need monitoring skills to control and destroy mosquito breeding ground. We need expertise to help us”.* (FGD-R4-male adult).

It was also pointed out that eliminating the mosquito breeding ground is a challenge which needs expertise at community level as insisted by one respondent who said that,

“*Eliminating the mosquito breeding grounds, is not an easy task, and seeking the help of experts is necessary to prevent them from multiplying. Even though we cannot remove or prevent mosquitoes from breeding, we can be taught how to eliminate their breeding grounds. Our knowledge is limited to maintaining a clean environment and using mosquito nets; we don’t have much expertise in eradicating mosquitoes”.* (FGD-R1-female adult).

Regarding the community approach on mosquito vector control, there were various opinions pointed out in terms of incentives needed for control mosquito in the environment. The opinion includes having village meetings with environmental sanitation education messages so that everyone could understand and actively participate as narrated by one respondent who said:

“*In my opinion, the first and foremost thing to do is to provide education. People need to be educated on maintaining a clean environment, including how to keep pools of stagnant water clean. We need more education on this topic.”* (FGD-R3-female adult).

A need for vector control interventions was further added by one respondent that,

“*As far as I understand, our current knowledge does not equip us to repel mosquitoes since I reside near a cowshed and ponds that provide my livelihood. If I were asked to fill them up, it would bring trouble to my family. Therefore, we are asking for a seminar to teach us how to remove mosquito breeding grounds. It is possible that, there is a larviciding which we can sprayed while continuing with our activities*”. (FGD-R7-female adult).

According to another respondent, he insisted the need for chemicals for killing mosquitoes as pointed out below.

“*I am requesting to have chemicals to kill mosquitoes because we cannot destroy ground water such as ponds as they are used to get drinking water and water for domestic use”.* (FGD-R1-male adult)

### Perceptions regarding women participation in vector control activities

The findings have revealed that majority of the respondents had a positive perception towards and accepted the idea of establishing and sustaining the ‘women in vector control’ groups at the community level. Respondents agreed that these groups of women would help educate people on the importance of keeping their environment clean and the use of various mosquito control interventions. During the group discussion, male respondents recognized the huge contribution which women do to control mosquitoes such as cleaning the surrounding environment and remove wastes as added by a male respondent who said:

“*Women participate specifically on cleaning the environment and ensuring mosquitos bed net are used in the household.*” (FGD-R3-male adult)

Among the activities done by men to control vectors includes cleaning the environment, buy mosquito nets and use mosquito nets. According to a male respondent who said:

*“As men we have great participation to control mosquito, like building house, and buying net that allow us to control mosquitoes in households.*” (FGD-R4-male adult)

Women recognize the unique ability of passing information passionately and persuasively to other people. They also understand and recognize their efforts in mosquito vector control in their families as narrated by a female respondent that,

“*Women play a great significant role than men in preventing mosquitoes within households. For example, women are the ones who usually wake up at night to ensure that children are protected from mosquito bites.”* (FGD-R5-female adult)

The women’s gender role was further narrated by one participant during focused group discussion that;

*“As I understand, women are very important because family matters are our responsibility. We are responsible for home environment and children.’’* (FGD-R5-female adult)

There was a general support for the idea of establishing a group of women dedicated to educating others about mosquito control. The group of women in vector control will help to educate their fellow and the community on general regarding vector control activities. This initiative was supported by majority of respondents. All respondents stated that they are ready to accept, support and collaborated with the group of women who will be established to serve as trainer of their fellow in the community.

On the other hand, few male respondents were sceptical of the initiative of establish and sustain the ‘women in vector control’ groups at the community level. They believed that women occupy a subordinate social position in society with little or no privilege and authority and thus making it hard for them to participate in the public activities including mosquito vector control. As narrated by one male participant who said that,

“*Women will face a challenge especially when providing education to households, some of people will be difficult to understand them.”* (FGD-R3-male adult)

### Women trainers of trainees (TOTs) empowerment

Majority of participants stated that they are ready to collaborate with the women trainers of trainees (TOTs) in their community and emphasized the need for more education to empower women in the fight against mosquitoes and mosquito-borne diseases. Empowering women involves providing them with incentives such as tools, education and support while engaging community-based activities. Shared responsibility between women and men was pointed to be important as stated by one respondent that,

*“In my view, a significant portion of our communities, particularly men, could benefit from provided education. They should be advised that domestic responsibilities are not the sole responsibility of women; there are other aspects where we need to collaborate and participate together. Therefore, cleanliness should be a shared responsibility” (FGD-R12-male youth-K).*

Women are seen as a powerful force, and using them for community education can be successful with proper training. They are seen as motivators for environmental cleanliness. They are crucial contributors to mosquito control due to their roles in managing households and implementing preventive measures as pointed out by one participant that,

*“… women are a powerful force, but also women are very understandable and can educate the community. We will reduce the burden on women and children”* (FGD-R9-female adult-K)

Focused group discussion with village and cultural leaders have revealed the importance of women to take the lead in provision of education to the community as narrated by one respondent that,

“*Maybe by establishing women groups to control mosquitoes and malaria through education, women can take the lead. They can motivate each other to purchase mosquito nets. Once they have acquired the knowledge, they will share it with others, and it can gradually disseminate throughout the entire village, so that everyone becomes aware of the issue of mosquitoes.*” (FGD-R4-village leader).

The cultural leaders highlighted the need respect and preservation of cultural norms in their communities while implementing the vector control activities.

### Community perceptions and support for women's participation in vector control activities

It was evident that this community will support the initiative of establishing the ‘women in vector control’ groups. Majority of respondents agreed that they will support them. Several reasons were stated with regard on why this group is important; such reasons include to prevent mosquito bites, provide education to the community and to deal with environment cleanness. On the other hand, women also support the establishment of women-led vector control groups for vector control activities in their community and agree that this group of women educators will be accepted.

It was clear that, the establishment of women groups for vector control activities in their community will be helpful as narrated by several respondents,

*“….. would be successful as it educates the entire community.”* (FGD-R1-female youth).

The contribution of women in house cleanness was further narrated by another respondent that;

*“Women are usually in charge of the household environment. They are typically the ones who are responsible for cleaning and maintaining the household on daily basis*”. (IDI-R1-female Mwagagala village)

Community support was vivid during the discussion as village and sub-village leaders mentioned that they will introduce the women trainers in their community for them to work smoothly. Majority of respondents also indicated that the community would support and sustain this group of women through provision of incentives such as transport, allowances and food.

Respondents had different views if they will be required, to donate a little money to help the women for the purpose of enhancing their visits to the households to provide education on how to control mosquitoes. Among the supports which were pointed out by village leaders including raising funds so that the women will get incentives while involving in vector control activities, sensitizing the community using whistles to be attention to be trained on vector control activities, to create bylaws for revenue collection to support women. Also, to engage the government, non-governmental organizations and politicians to support this group of women as narrated by one respondent who said:

“*One way is to raise funds so that they can receive allowances, and they can form groups to lend money for their activities.*” (FGD-R2-village leader)

### Anticipated challenges regarding establishing and sustaining women-led vector control initiative

During the discussion, several challenges mentioned were similar across the sites. They included lack of common understanding specifically to people who had not yet heard about the aim of establishing this group of women in vector control. Also, lack of incentives such as salary while participating in vector control activities at their community. Again, limited time to engage themselves in agricultural activities while participating in vector control activities was among the anticipated challenges as narrated by several respondents who said:

“*They can fail to implement activities because of lacking salary.*” (FGD-R2-male adult. “*As previously mentioned, we are all peasants, and therefore, we may not have enough time to participate during the rainy season.*” (FGD-R8-male adult)

Regarding resistance to change, another respondent added that;

“*After understanding the lesson, some people can be motivated while others may be hesitant to learn if it is difficult”.* (IDI-8-female at Mwagagala village)

Inadequate support and discouragement from their husbands were also mentioned as anticipated challenges. Attending competing interest activities such as farming and at the same time engaging in community education about mosquito vector control activities as pointed out by one female respondent who said:

*“As a woman coming from my family, there are potential challenges we may face in this group. If my husband asks me the intention of the group and I mention that we are learning how to prevent mosquitoes, he may discourage me by pointing out that mosquitoes have existed for centuries, and we cannot combat them. As a result, he may prohibit participating in these groups. Furthermore, the community may also dismiss me, claiming that I am incapable of battling mosquitoes, and hence, I will have no meaningful contribution to make. However, in my family, when I bid them farewell, others will suggest that we should go to the farm, to fight against mosquitos we will use mosquito nets, and set a fire to repel mosquitoes. It is important to provide education to the whole community regarding the working women’s schedule and their duties, and to gather their feedback to assess the quality of their duties*.*”* (FGD-R6-female adult)

Other challenges mentioned including that some people will refuse to give them cooperation or follow the advice given or pretending to be busy and not paying attention to this group of women expected to be established to serve the community. Resistance to change due to traditional beliefs, lack of tools and incentives, and language barrier. Lack of participation from young people, especially when parents are not present at home, negative responses, from the community member, lack of transportation tools, lack of collaboration with husbands/partners, resistance to changing habits among community members as sated by one respondent that,

*“Challenges are always there. In my opinion, if there’s a group of women going from house to house, in some towns, people may not understand; they may think you’re just making noise. One person may pretend to be busy, and another may not follow what you’re educating them about”, (FGD-R6-female adult-K).*

The beliefs and habits of viewing malaria as a common disease were also pointed out as narrated by one respondent that,

*“One challenge from people’s is traditional beliefs and habits of viewing malaria as a common disease. Even with education, they may dismiss it, claiming they are used to the disease and can recover by taking a mixed drug” (FGD-R11-male youth-K).*

Other challenges mentioned including the behaviour of being mocked by community members as pointed out that,

*“If you visit households of your neighbours, there are challenges and possibility of being ridiculed and laughed at, however, that may not limit your work.”* (FGD-R2-female adult)

### Possible solutions mentioned by participants

Lack of proper education and men engagement that need to be addressed through community meetings to have a common understanding in the community as narrated by some respondents who said:

*“In order to address challenges that we might encounter during fieldwork we need seminars so that to understand on how to control malaria, this will help in addressing all doubt that might arise.”* (FGD-R1-female adult). “*Men should be educated about this programme so that they can allow us to participate in educating others.*” (FGD-R2-female adult). “*There is the need of gathering to educate people about mosquito control.*” (FGD-R3-male adult)

*“We believe that women will not be prevented from participating in educating other households about mosquito control.*” (FGD-R2-male adult)

“*This work should involve paying salary to women so that the money offered facilitate other activities.*” (FGD-R1-male adult)

“*We require execution, and it is crucial for both men and women to have a mutual understanding and come to an agreement regarding this activity.”* (FGD-R4-male adult). *“Education should be provided to both women and men, we also need that education”,* FGD-R6-male adult. “*Women and men should be educated on how to control mosquitoes”,* FGD-R3-female adult.

## Discussion

The present study has provided valuable insights on community perceptions and support for establishing and sustaining the women-led groups for mosquito vector control at the community level in Misungwi and Ilemela. The findings have revealed the community acceptance and positive perceptions towards engaging women as champions of vector control. These findings aligns with a previous study, which documented the contribution of women as key change agents in delivering health interventions such as vector-borne diseases control (Mungall-Baldwin, 2022).

The findings on the community understanding of mosquitoes and breeding sites have demonstrated a basic understanding of mosquito behaviours, potential breeding sites and their medical importance as vectors of diseases like malaria, lymphatic filariasis and dengue. These findings are supported by previous review (Duval et al., 2023) and a previous study conducted in Babuque island in Guinea Bisau (Hutchins et al., 2020). They also mentioned common mosquito breeding grounds such as stagnant water, water storage containers and puddles, which aligns with known mosquito ecology (Egid et al., 2022). The findings of this study also concur with a previous study conducted in rural upper valleys of Nepal, which mentioned the environment around the house related to an increase in mosquitoes that cause malaria provided that there are ditches or potholes, dirt and stagnant water where mosquitoes can breed (Awasthi et al., 2022)*.*

However, there were mixed views on whether domestic water storage containers could serve as potential breeding grounds. While some participants recognized that uncovered and stagnant water could lead to mosquito breeding, others believed that water stored in clean or grassy areas would not pose a risk. The partial understanding regarding mosquito breeding sites has been documented by a previous study conducted in Alabama (Morse et al., 2019). This suggests that more targeted education on how improper water storage contributes to mosquito proliferation could help to address these misconceptions. These varying perceptions underline the importance of providing more targeted education on the critical role that improper water storage plays in mosquito proliferation. Community engagement as trainers of trainees (TOTs) in mosquito vector control programmes have proven to be invaluable (Carboni et al., 2023). In this context, the role of the community is going to be streamlined to the women as TOTs in mosquito vector control where they will be working directly with the local community to address the dispelling misconceptions and providing essential education. Additionally, they will help to bridge the gap in local knowledge about the risks of improperly stored water and thereby transforming local attitudes and behaviours, ultimately helping to reduce the spread of vector-borne diseases.

Regarding the risks associated with mosquito bites, participants were aware of health risks posed by mosquito bites particularly to pregnant women and children. They mentioned health conditions such as fever, body weakness and vomiting were associated with mosquito bites. This presents an opportunity for health programmes to broaden the scope of education to include all mosquito-borne diseases, especially in areas where multiple mosquito vectors are present.

There was a strong consensus among participants that environmental cleanness is key towards controlling mosquito breeding sites. Highlighted activities included clearing of bushes, filling water holes and cutting grasses. These suggestions align with integrated vector management (IVM) strategies which emphasize environmental management as a crucial component in controlling mosquito (Beier et al., 2008). The importance of IVM has also been highlighted by a previous study conducted in Zambia (Chanda et al., 2008) According to the World Health Organization (WHO), the use of a range of interventions in combination and engagement of local communities has also been emphasized (WHO, 2004). In the present study, the importance of using insecticide-treated nets (ITNs) was also recognized, even though, during warm weather, the nets were not used effectively leaving people at risk of getting mosquito bites. The community emphasized the need for ongoing education to raise awareness on mosquito control.

A major theme that emerged was the potential of women-led groups to foster greater community involvement in vector control activities and sustainability. Participants proposed forming a group of women TOTs to educate other on mosquito control align with previous successful public health interventions where community health workers played key roles in health education and behaviour change as evidenced by a study conducted in Guyana (Carboni et al. 2023) and Cameroon (Mbako, 2017). However, when this approach is streamlined to women, it could be a valuable strategy for increasing community ownership for mosquito control in rural areas where women are primarily caregivers and safeguarding their families’ health within households. In the present study, majority of respondents acknowledged the role played by women in household cleanliness and ensure that their family members adhere to use of mosquito bed nets and other preventive measures. Interesting, one notable aspect of the discussion was the broad support for establishing and sustaining women-led groups focused on mosquito vector control. Both men and women recognized the perceived potential impact of such groups in educating their community on proper mosquito control practices. However, it is crucial to understand the strategies that enhance women’s participation in order to narrow the gender gap in mosquito control activities (Donner et al., 2017).

Another key theme identified in the present study is the women’s ability and strength to communicate persuasively and motivate others. Regarding the perceived perceptions of Women strength in community engagement of vector control was vivid as they were delighted of their contributions to household mosquito control, keeping their families safe from malaria through environmental hygiene, bed net use, and ensuring timely medical care for their children. This recognition of women’s leadership in household vector control supports the broader idea of formalizing their role within the community through establishment of women-led vector control groups. The findings of this study are contracting with the previous evidence which states that, at home women continue to bear most unpaid tasks related to reproduction and caregiving, which limits their capacity to engage in activities outside the household including economic decisions (Navy & Bunheng, 2009). These groups could get employment opportunities and therefore challenge earlier research, which suggested that women’s roles within the household are still largely confined to tasks.

While most respondents were supportive, a few male participants expressed scepticism, citing women traditionally subordinate social roles. These concerns reflect gender dynamics that may pose challenges to the success of women-led initiatives. They are reflective of broader societal attitudes that often place limitations on women’s participation in leadership and decision-making roles outside the household. Despite these reservations, the overall sentiment remained positive, with respondents acknowledging the need for women’s involvement and education to improve vector control outcome.

The cultural and village leadership endorsement of women-led vector control initiative predicts the potential for acceptance, sustainability and success in a traditionally male–male-dominated sphere of public activity. They emphasized the need for cultural norms respect and preservation while implementing vector control activities. Respondents also called for both financial and logistical support, both gender involvement and men support to their wives.

## Conclusion

Communities accept and support the idea of establishing and sustaining ‘women-led vector control’ groups to control and eliminate VBDs. The findings of this study reflect a community-driven approach of using empowered group of women to address the challenges associated with mosquitoes and malaria, with an emphasis on education, prevention and collaboration. The findings have revealed that these communities are aware of the health risks posed by the mosquito bites. It also highlights the importance of environmental cleanness and role of education in community to combating malaria to the community. The participants emphasized the importance of local initiatives in addressing mosquito-borne diseases control and expressed their willingness to collaborate and contribute to solutions that can have a positive impact on their community’s health. Generally, the discussion highlights the importance of community engagement, gender-specific roles and collaboration with local authorities in the fight against malaria.

## Data Availability

All analysed data involved in this manuscript are included in this manuscript.
